# Silencing of *PhLA*, a *CIN*-*TCP* gene, causes defected petal conical epidermal cell formation and results in reflexed corolla lobes in petunia

**DOI:** 10.1186/s40529-020-00300-7

**Published:** 2020-09-17

**Authors:** Hsiao-Wei Chen, Po-Lun Lee, Chun-Neng Wang, Hui-Ju Hsu, Jen-Chih Chen

**Affiliations:** 1 Institute of Biotechnology, National Taiwan University, Taipei, 106 Taiwan, ROC; 2 Agricultural Biotechnology Research Center, Academia Sinica, Taipei, 115 Taiwan, ROC; 3 Department of Agronomy, National Taiwan University, Taipei, 106 Taiwan, ROC; 4 Institute of Ecology and Evolutionary Biology, National Taiwan University, Taipei, 106 Taiwan, ROC; 5 Department of Life Science, National Taiwan University, Taipei, 106 Taiwan, ROC

**Keywords:** CIN-TCP, LANCEOLATE, Petunia, Virus-induced gene silencing, Conical epidermal cell, Petal curvature

## Abstract

**Background:**

TCP-domain proteins, plant specific transcription factors, play important roles in various developmental processes. CIN-TCPs control leaf curvature in simple leaf species while regulate leaf complexity in compound leaf species. However, the knowledge was largely based on findings in few model species. To extend our knowledge on this group of proteins in Solanaceae species, we identified a *CIN*-*TCP* gene from petunia, and studied its functions using virus-induced gene silencing (VIGS).

**Results:**

Consistently, silencing of *CIN*-*TCPs* increases complexity of tomato leaves, and enhances leaf curvature in *Nicotiana benthamiana*. However, in petunia (*Petunia hybrida*), silencing of petunia *LA*, a *CIN*-*TCP*, through VIGS did not obviously affect leaf shape. The silencing, however, enhanced petal curvature. The event was associated with petal expansion at the distal portion where epidermal cell size along the midribs was also increased. The enlarged epidermal cells became flattened. Although shapes of *PhLA*-silenced flowers largely resemble *phmyb1* mutant phenotype, *PhMYB1* expression was not affected when *PhLA* was specifically silenced. Therefore, both PhLA and PhMYB1 are required to regulate flower morphology. In corolla, *PhLA* and *miR319* deferentially express in different regions with strong expressions in limb and tube region respectively.

**Conclusions:**

In conclusion, unlike *LA*-like genes in tomato and *N. benthamiana*, PhLA plays a more defined role in flower morphogenesis, including petal curvature and epidermal cell differentiation.

## Background

Plant stature is controlled by tightly regulated developmental processes where TCP transcription factors play important roles (Dhaka et al. 2017; Rosin and Kramer 2009). A genome wide study on transcription factors of tobacco (*Nicotiana tabacum*) revealed that the expansion of TCP family is strongly associated with the evolutionary diversity in Solanaceae (Rushton et al. 2008). TCP-domain proteins participate in various developmental processes, including floral symmetry, axillary bud outgrowth, leaf curvature, shape avoidance, and flowering (Doebley et al. 1997; Luo et al. 1999; Nath et al. 2003; Silva et al. 2019; Zhou et al. 2018). However, studies of TCP proteins are largely restricted to few model organisms. To extend our knowledge to how TCP proteins contribute to the diverse forms of Solanaceae species, we identified the ortholog of tomato (*Solanum lycopersicum*) *LANCEOLATE* (*LA*), a *CIN*-like TCP gene, from petunia (*Petunia hybrida*), and named it *PhLA*. Its function on organ shape regulation in petunia was investigated using virus-induced gene silencing (VIGS).

TCP proteins, containing a conserved non-canonical basic-Helix-Loop-Helix (bHLH) domain, were first defined after three funding members, TEOSINTE BRANCHED1 (TB1) from maize (*Zea mays*), CYCLOIDEA (CYC) from *Antirrhinum* (*Antirrhinum majus*) and PROLIFERATING CELL FACTORS (PCFs) from rice (*Oryza sativa*) (Cubas et al. 1999). The conserved domain, named TCP domain, composed of 59 amino acids, is required for DNA binding and the interaction between proteins (Cubas et al. 1999). Based on differences in their TCP domains, this family of transcription factors can be divided into two groups, with PCFs in class I and TB1 as well as CYC in class II (Hilernan and Preston 2009; Howarth and Donoghue 2006). PCF1 and PCF2 were found to bind the promoter of the *PROLIFERATING CELL NUCLEAR ANTIGEN* (*PCNA*) gene and promote cell proliferation (Kosugi and Ohashi 1997). TB1 prevents axillary bud outgrowth (Cubas et al. 2007; Doebley et al. 1997), and CYC along with another TCP protein, DICHOTOMA (DICH), establish zygomorphic symmetry of *Antirrhinum* flowers (Galego and Almeida 2002; Luo et al. 1996; 1999). The origin of TCP proteins can be tracked back to the ancestor of land plant lineage, and is considered to be important for evolution of multicellularity (Floyd and Bowman 2007). Duplication and diversification during evolution made the family become larger and associated to various floral traits (Chapman et al. 2008; Mondragon-Palomino and Trontin 2011).

The class II members can be further divided into the ECE clade, including CYC and TB1, and the CIN clade, including CINCINNATA from *Antirrhinum* and TCP4 from *Arabidopsis* (*Arabidopsis thaliana*) (Hilernan and Preston 2009). CIN suppresses cell division during leaf lamina development in *Antirrhinum*, and therefore control leaf curvature (Nath et al. 2003). While suppressing cell division, CIN promotes growth of *Antirrhinum* petal lobes (Crawford et al. 2004). Eight *CIN* class genes were distinguished from a total of 24 *TCP* genes in *Arabidopsis* genome (Martin-Trillo and Cubas 2010). Among them, *TCP2*, *3*, *4*, *10* and *24* are post-transcriptionally regulated by *miR319. jaw*-*D* mutations resulted in transcription suppression of these genes due to ectopically expressed *miR319a*, and led to phenotypes largely resembling those of *cin* mutants in which crinkly leaves particularly on the margins were exhibited (Palatnik et al. 2003). Mutations on one *CIN*-*TCP* alone in *Arabidopsis*, however, exhibit very mild phenotypic changes (Schommer et al. 2008); however, when expressions of more and more members of this group were suppressed, dramatic synergic effects in leaf development were observed and led to extremely large and serrated leaves (Efroni et al. 2008). These results suggest a functional redundancy among *CIN*-*TCP* genes in *Arabidopsis*. Loss of CIN-TCP activity affects leaf curvature in simple leaf species while suppression of the activity enhances the complexity of tomato leaves, a compound leaf species (Ori et al. 2007). On the other hand, misexpression of *LA* due to mutation on its *miR319* recognition site resulted in the conversion of tomato compound leaf into simple one (Ori et al. 2007). It was hypothesized that this group of proteins promotes leaf maturation and is important for timing control of the transition from the morphogenetic phase to the differentiation phase. Indeed, the alteration of *LA* transcript abundance was associated with the timing of growth arrest and maturation of tomato leaves (Shleizer-Burko et al. 2011). Interestingly, leaf size can also be controlled by manipulation of the activation time of CIN-TCPs during leaf maturation in *Arabidopsis*. Precocious activation of a *miR319* insensitive version of TCP4 led to generation of miniature leaves. In contrast, delayed activation of CIN-TCPs exhibited expanded leaves (Efroni et al. 2008). The findings suggest that the dynamic spatial and temporal controls of this maturation program are a reason for diverse leaf sizes and shapes.

Functions of CIN-TCPs in controlling leaf morphogenesis have been studied in depth while their roles in flower development are less understood. However, it was shown that misexpression of a *miR319* insensitive form of *TCP4* (*mTCP4*), but not a wild type copy of *TCP4*, suppressed petal growth (Nag et al. 2009), while reduction of CIN-TCP activity because of mutations in *CIN*-*TCP* genes or over-expression of an artificial repressor protein, TCP5-SRDX, where the TCP5 was fused with a strong transcriptional repressor domain, SRDX, yielded serrated and wavy petals (Koyama et al. 2011). The same chimeric TCP-repressor strategy was applied to *Cyclamen persicum* to create ruffled petals for this commercially important floriculture plant (Tanaka et al. 2011). The results suggest that like their functions in leaf development, CIN-TCPs also negatively regulate cell division during petal development. However, cell proliferation was reduced in petal lobes of *Antirrhinum* when *CIN* was mutated (Crawford et al. 2004). Therefore, further studies will be needed to clarify how CIN-TCPs regulate petal development.

We took the advantage of a rapid functional analysis technique, VIGS, to characterize the effects of knocking down *LA*-like genes on leaf and flower development in petunia, tomato, and *N. benthamiana*. A TRV-based gene silencing system that utilizes chalcone synthase (*CHS*) gene as gene silencing marker to distinguish floral tissues with target gene silenced (white flowers) to those without target gene silenced (purple flowers) has been developed for petunia (Chen et al. 2004). Using the system, we have successfully knocked down expression of *PhLA* and characterized its functions in flower shape regulation in petunia. The effects of *LA*-like gene silencing were also compared among petunia, tomato, and *N. benthamiana*.

## Materials and methods

### Plant material and growth condition

Petunia (*Petunia *×* hybrida* cv. Primetime Blue) seeds were produced by Goldsmith Seeds (Gilroy, CA, USA), and W115 seeds were kindly provided by Dr. Tom Gerats, Radboud University, Netherlands. *Solanum lycopersicum* cv. 5915 and *Nicotiana benthamiana* seeds were gifts from Dr. Chiu-Ping Cheng, National Taiwan University, Taiwan. Plants were grown in a culture room at 23 ± 3 °C under 16 h light/8 h dark cycles.

### Phylogenetic analysis

Phylogenetic analysis of *PhLA* coding sequence with sequences encoding other TCP domain proteins was conducted using MAGA 5.0 by the maximum likelihood (ML) method with bootstrap value calculated from 1000 replicates. The partial sequence information for *PhLA* was first obtained from a petunia EST collection (accession number CV298738), which contains the 5′ half of the transcript, and rapid amplification of DNA ends PCR was used to extend the 3′-end sequence. The coding sequences were translated into amino acid sequences for alignment and then the aligned sequences were shifted back to nucleotide sequences for phylogenetic analysis. Only the first two positions of codons were used in the analysis. Genebank accession numbers of sequences used in the analysis are: *PhLA* (), *SlLA* (EF091571), *SlTCP3* (EF091574), *SlTCP10* (NM_001247647), *AmCIN* (AY205603), *AmCYC* (AY316729), *AmDICH* (AF199465), *AtTCP4* (NM_112365), *AtTCP2* (NM_117950), *AtTCP24* (NM_102760), *AtTCP5* (NM_125490), *AtTCP17* (NM_120889), *AtTCP13* (NM_111082), *AtTCP1* (NM_001160982), *AtBRC1* (NM_112741), *AtBRC2* (NM_105554), *ZmTB1* (ZMU94494). Here, the first two letters of gene symbols represent the organisms in which they are *Am* for *Antirrhinum majus*, *At* for *Arabidopsis thaliana*, *Sl* for *Solanum lycopersicum*, and *Zm* for *Zea mays*.

### VIGS of *PhLA*

Tobacco rattle virus–based VIGS vectors, pTRV1 and pTRV2, were kindly provided by Dr. Dinesh-Kumar, Yale University, USA (Liu et al. 2002). The pTRV2 was previous modified to include a *CHSJ* fragment as a silencing reporter (Chen et al. 2004), and the derived construct, named pTRV2 *CHS*, was used as a control in silencing experiments.

**Fig. 1:**
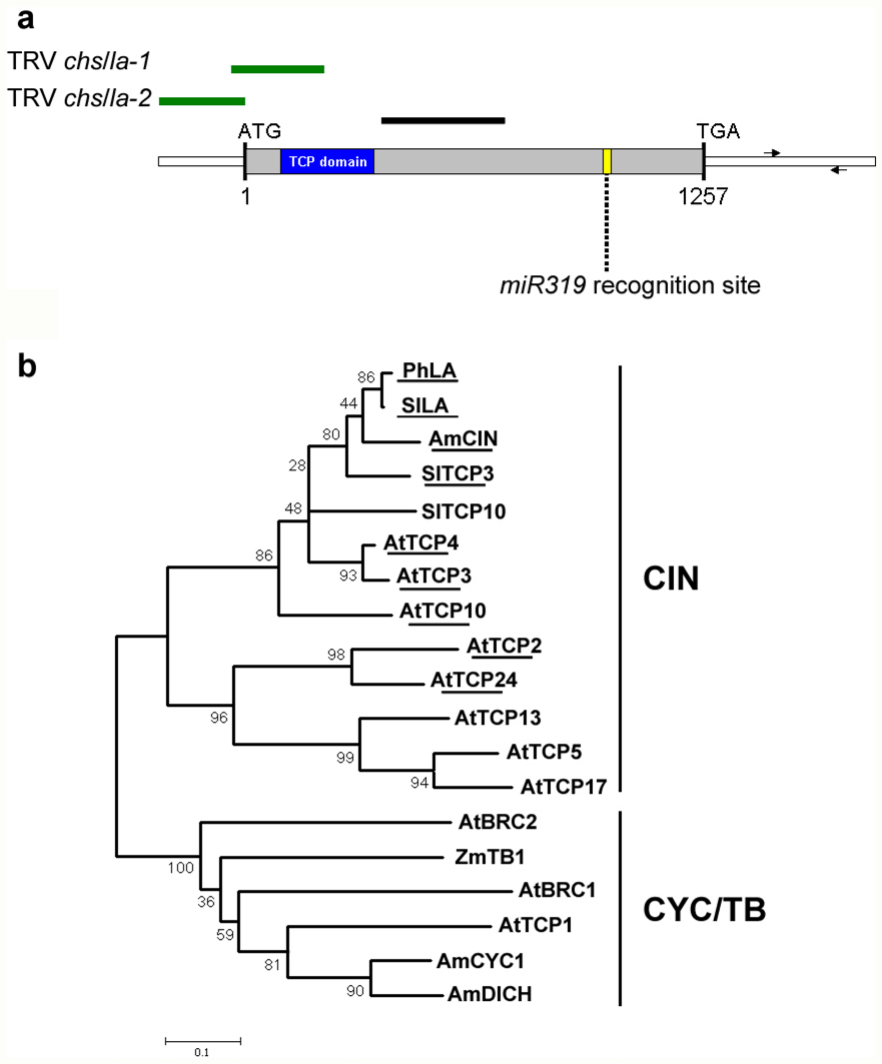
Schematic representation of *PhLA* cDNA and phylogram of its sequence with other reported TCPs. **a** cDNA structure of *PhLA*. Relative positions of *PhLA* open reading frame and the 5′ and 3′ UTR are indicated. The blue box marks the region encoding TCP domain and the yellow line shows the location of *miR319* recognition site. Two fragments used for VIGS analysis are indicated in green, and the black line shows the probe region for in situ hybridization. Arrows show location and direction of primers used to measure transcript abundance. **b** Phylogenetic tree for *TCP* genes. Class II TCPs were divided into CYC/TB subclade and CIN subclade. *PhLA* was grouped in CIN subclade and is most closely related to *SlLA* in all genes analyzed. The transcripts containing a *miR319* recognition site are underlined. For sequences used in this study, their GenBank accession numbers are listed in “Materials and methods”

Two *PhLA* cDNA fragments were used to knockdown expression of *PhLA*. A fragment from 28 bp before start codon (− 28) to 219 bp after the start codon (+ 219) was cloned into pTRV2 *CHS* to create pTRV2 *CHS*/*LA*-*1*. This fragment contains a conserved sequence region encoding TCP domain and was designed to knockdown expression of more than *PhLA*. The other fragment from − 235 bp to − 5 bp was also cloned into pTRV2 *CHS* to generate pTRV2 *CHS*/*LA*-*2*. This fragment is at the 5′-UTR of the *PhLA* transcript to ensure a specific gene silencing on *PhLA* (Fig. 1a).

VIGS was carried out using *Agrobacterium*-mediated transfection as described by Chen et al. (2004). Seedlings at 3–4 true leaf stage for petunia as well as *N. benthamiana*, and at the first true leaf stage for tomato cv. 5915 were used for all gene silencing experiments.

### Real time RT-PCR analysis

Total RNA of petunia was extracted using the TRIzol reagent (Invitrogen Corporation, Carlsbad, CA) based on manufacturer’s instruction. Before cDNA synthesis, total RNA was treated with TURBO DNA-free kit (Invitrogen Corporation, Carlsbad, CA) to remove any contaminating DNA. First-strand cDNA was synthesized using MMLV reverse transcriptase (Invitrogen Corporation, Carlsbad, CA), and used as template for real time PCR. PCR primers were designed outside of the regions used for VIGS to prevent cross detection to TRV2 that contains target gene fragment. Primers for amplifying *PhLA* transcripts were 5′-CGAGTCTAATTCAATGGCGTGC-3′ and 5′-AGACTTGTCATGCTGTTGCC-3′. Primers for amplifying *PhMYB1* transcripts were 5′-GAGACATTCACAGACCTTTTGC-3′ and 5′-AACATAGCTGAATCTGAGGGTG-3′. *ACTIN* was used as internal control and primers for amplifying its transcripts were 5′-TTGTCCGTGACATGAAGGAA-3′ and 5′-TCGATGGCTGGAAGAGAACT-3′. The PCR products were sequenced to ensure the amplification of correct genes. Results were tested in at least two independent experiments, three samples and three replications. The relative expression of target genes was calculated by the 2^−ΔΔCT^ method and displayed as fold change when compared with the control group.

### In situ hybridization

The in situ hybridization protocol basically follows Wang et al. (2008) which is designed for step by step optimization for non model plants. Collected petunia inflorescences were fixed in ice cold fresh paraformaldehyde and dehydrated through ethanol/xylene series for later parafilm embedding. The embedded tissues were sectioned to 8 μm slides with microtome and hybridized with DIG-labeled RNA probes. The 346 nt probe was designed from 664 to 1009 of *PhLA* transcript (Fig. 1). The cDNA fragment corresponding to this region was cloned into pGEM-T easy between *Spe*I and *Nco*I sites. In vitro transcription was used to produce sense and antisense *PhLA* probes. For *miR319* detection, a locked nucleic acid (LNA) oligonucleotide probe was synthesized by substituting every third nucleotide with a LNA monomer (Exiqon), and the 5′-End and 3′-End of this probe were labeled with DIG.

### Scanning electron microscopy

Petunia flower segments were collected and fixed in FAA. Tissues were dehydrated through ethanol series to 100% acetone, then precede to critical point drying by HCP-2 (Hitachi Ltd, Japan) and ion coater (IB-2, Eiko Engineering, Japan) to sputter-coat with gold/palladium. The coated tissues were then observed under Scanning Electronic Microscope.

## Results

### Isolation of petunia *LANCEOLATE* from petunia and sequence analysis

A putative *CIN*-*TCP* sequence (accession number CV298738) was identified from a petunia floral expressed sequence tag (EST) database using the tBLASTn tool with CINCINNATA (CIN) amino acid sequence as the query. A 247 bp partial sequence, which contains part of conserved sequence encoding TCP domain, of this EST was then amplified from petunia floral cDNAs and sequenced. The fragment was then cloned into the TRV2 vector containing a *CHS* cDNA fragment for VIGS (Fig. 1a). To obtain the full length coding sequence (CDS) of this *CIN*-*TCP*, RACE-PCR was used and a fragment of 1961 bp containing a 1257 bp of the CDS was amplified (accession number). The nucleotide sequence of this CDS shares 81 to 82% identities with other *LANCEOLATE* CDS sequences while its deduced amino acid sequence shares 78% identities with LANCEOLATE from other Solanaceae species, including tomato (accession number ABM65599), *Capsicum annuum* (ADN51990), *Solanum tuberosum* (ADN51992), and *Solanum melongena* (ADN51991); 59% identity with *Antirrhinum* CIN (AAO43102); and 47% identity with *Arabidopsis* TCP4 (AT3G15030). The sequence identities among reported LANCEOLATE proteins from Solanaceae species are around 80%, and therefore, we named the petunia CIN-TCP Petunia LANCEOLATE (PhLA). The transcript of *PhLA* also contains a *miR319* recognition site at positions 984 to 1104 (Fig. 1a). A phylogenetic analysis of known class II *TCPs* using bootstrap consensus for maximum likelihood (ML) revealed that *PhLA* is most closely related with tomato *LANCEOLATE*, and with other *miR319* regulated *CIN*-*TCPs* (Fig. 1b).

### Silencing of *LA*-like genes altered leaf morphology in *N. benthamiana* and tomato, but not in petunia

**Fig. 2:**
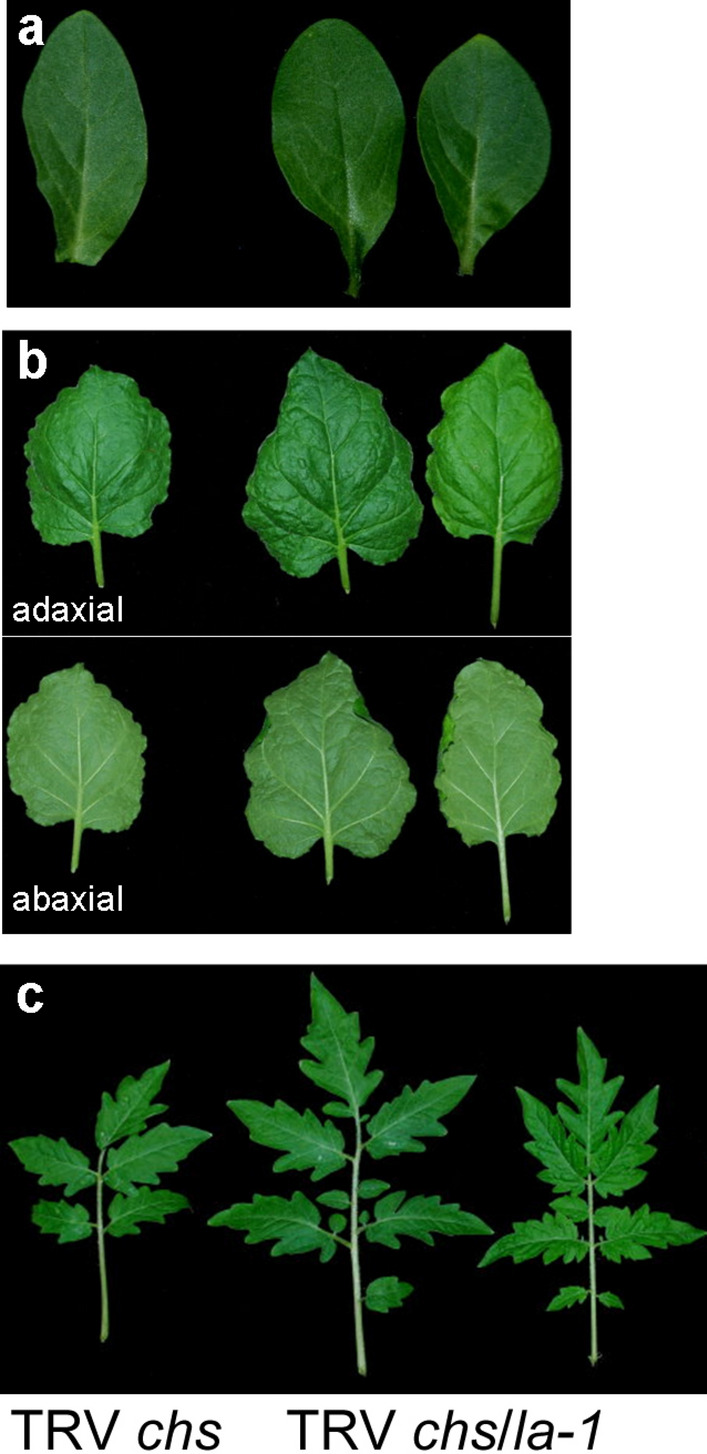
Comparison of effects of silencing *LA*-like genes on leaf morphology in petunia (*Petunia hybrida*), *Nicotiana benthamiana*, and tomato (*Solanum lycopersicum*). **a** Petunia leaves with or without *LA*-like gene silenced. No obvious morphological change on petunia leaves is observed. **b** *N. benthamiana* leaves with or without *LA*-like gene silenced. Leaf curvature is increased when *N. benthamiana* plants infected with TRV carrying a fragment of *PhLA* sequence. Abaxial side of the leaves shows outgrowth on the leaf margins. **c** Tomato leaves with or without *LA*-like gene silenced. The fourth leaves of tomato plants were compared

**Fig. 3:**
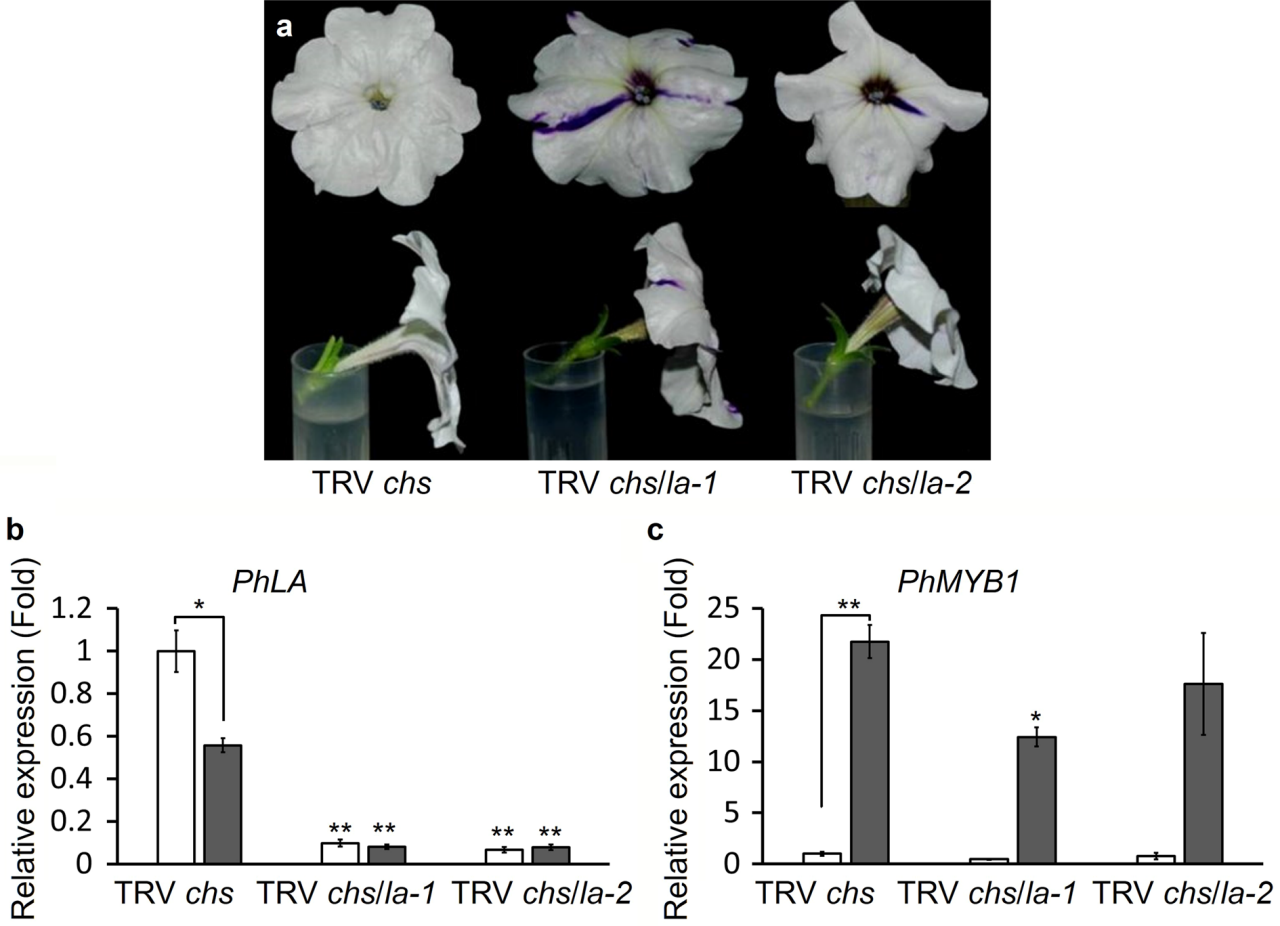
Effects of silencing *PhLA* on petunia flower. **a** Petunia flowers with or without *PhLA* silenced. White flowers of petunia plants infected with either TRV *chs*/*la*-*1*, which contains a conserved fragment of *PhLA* cDNA, or TRV *chs*/*la*-*2*, which contains a gene specific fragment of *PhLA* cDNA, shows strongly reflexed corolla lobes. **b** Relative expression of *PhLA* in leaves (gray bars) or white flowers (white bars) of petunia plants infected with TRV *chs*, TRV *chs*/*la*-*1*, or TRV *chs*/*la*-*2*. **c** Relative expression of *PhMYB1* in leaves (gray bars) or white flowers (white bars) of petunia plants infected with TRV *chs*/*la*-*1* TRV *chs*, TRV *chs*/*la*-*1*, or TRV *chs*/*la*-*2*. Transcript abundances of *PhLA* and *PhMYB1* are relative to those in white flowers of the TRV *chs* group. Expression of *ACTIN* gene was used as an internal control. *Indicates significant differences to the control groups (*P < 0.05, **P < 0.005)

To examine the function of *LA*-like genes in petunia, we inoculated young petunia seedlings with TRV containing *CHS*/*LA*-*1* tandem fragment (TRV *chs*/*la*-*1*) using *Agrobacterium*-mediated infection. Virus-induced silencing of *LA*-like genes in petunia, however, did not result in leaf lamina overgrowth (Fig. 2a), which was observed in *Antirrhinum*, *Arabidopsis*, and tomato when activity of their CIN-TCPs were repressed (Nath et al. 2003; Ori et al. 2007; Palatnik et al. 2003). Since it was shown that lose of tomato LA activity led to enhanced leaf complexity, the same TRV *chs*/*la*-*1* was used to infect tomato seedlings, and indeed, in the virus-infected tomato plants, the complexity of tomato compound leaves increased presumably due to silencing of *LA*-like genes because nucleotide sequence of the *la*-*1* region shares 91% identity with tomato *LA* (Fig. 2c). Silencing of *LA*-like genes by inoculation of the TRV *chs*/*la*-*1* into *N. benthamiana* also resulted in enhanced leaf curvature, particularly at the margins (Fig. 2b). Because silencing of *PhLA* did not result in visible morphological changes in leaves, transcript abundance of *PhLA* in leaves was examined using real-time RT-PCR, and the results indicate a significant downregulation of *PhLA* was in leaves of TRV *chs*/*la*-*1*, and TRV *chs*/*la*-*2* infected petunia plants (Fig. 3b). Therefore, the lack of morphological changes in petunia leaves was not due to inefficient gene silencing in leaves.

### Silencing of *LA*-like genes enhanced corolla curvature in petunia

Though silencing of *LA*-like genes in petunia did not cause any obvious changes in leaf development, clearly, it resulted in enhanced petal curvature (Fig. 3a). White flowers, indicating successful silencing in both *CHS* and *LA* genes, of petunia plants infected with TRV *chs*/*la*-*1* were strongly curved backward (Fig. 3a). Because *PhLA* fragment in TRV *chs*/*la*-*1* is in a conserved region, it is very possible that in addition to *PhLA*, other *LA*-like genes were silenced. In order to understand whether the change in PhLA activity alone will be effective enough to affect the petal curvature, a gene specific sequence was amplified from 5′-UTR of *PhLA* and cloned into pTRV2 (TRV *chs*/*la*-*2*) for VIGS since un-translated regions are generally gene specific and the use of 5′-UTR also limited silencing of non-targets through transitive RNA silencing (Xie and Guo 2006). Indeed, white flowers of petunia plants infected with TRV *chs*/*la*-*2* also exhibited similar shapes to those infected with TRV *chs*/*la*-*1* (Fig. 3a).

*N. benthamiana* flowers infected with TRV *chs*/*la*-*1* also curved slightly backward (Additional file 1: Fig. S1). Flowers from tomato plants infected with TRV *chs*/*la*-*1*, however, did not show enhanced petal curvature (data not shown).

The petal presentation in *PhLA* silenced flowers largely resembled the floral phenotype observed in *phmyb1* mutants (Baumann et al. 2007). Similar to MIXTA in *Antirrhinum*, PhMYB1, another R2R3 MYB transcription factor, controls the development of conical epidermal cells in petunia petals (Baumann et al. 2007). Mutations in *PhMYB1* affected epidermal cell differentiation in petals and resulted in flat epidermal cells. Interestingly, the defect in conical cell development also led to alteration in petal presentation (Baumann et al. 2007). Since both *PhLA* silencing and *phmyb1* mutants increased petal curvature, we examined whether the morphological changes caused by *PhLA* silencing was resulted from reduction of *PhMYB1* expression. Our results show that *PhLA* can be efficiently silenced in both leaves and flowers using either a conserved fragment (TRV *chs*/*la*-*1*) or a gene specific fragment (TRV *chs*/*la*-*2*). Silencing of *PhLA* in flowers did not significantly affect the transcript abundance of *PhMYB1* while silencing of *PhLA* in leaves using a conserved sequence fragment (TRV *chs*/*la*-*1*) resulted in downregulation of *PhMYB1* (Fig. 3c). When comparing transcript abundance of *PhLA* and *PhMYB1* between flowers and leaves, we found that transcripts of *PhLA* was significantly more abundant in flowers than those in leaves while transcripts of *PhMYB1* was significantly more abundant in leaves than those in flowers (Fig. 3b, c).

### Changes in petal lobe shapes in *PhLA* silenced petunia flowers

The reflexed petal curvature caused by *PhLA* silencing should be due to shape changes in corollas. We compared the length, width, and distance between sinuses of lobes between flowers infected with TRV *chs* (flowers with corolla limb curvature close to zero) and those infected with TRV *chs*/*la* (flowers with negative corolla limb curvature). Both length and width of *PhLA* silenced flower lobes were significantly longer; however, the distance between sinuses in these flowers were much shorter. The ratio of width/length was not changed; however, the ratio of distance between sinuses/length in *PhLA* silenced flowers was reduced from 1.7 to 1.4 (Table 1). The overall area sizes in both groups, however, were the same. These measurements indicate that the outer lobe areas of *PhLA* silenced corollas were slightly expanded while their inner lobe areas became smaller. Therefore, we also observed a larger overlapping area between two lobes of *PhLA* silenced corollas.

**Table 1:** Measurements of lobes of fully expanded white flowers from TRV *chs* infected and TRV *chs*/*la*-*1* infected plants

	n	Lobe width (W) (mm)**	Distance between sinuses (D) (mm)**	Lobe length (L) (mm)**	W/L	D/L ***	Area (mm^2^)
TRV *chs*	125	29.4 ± 2.2	24.2 ± 1.3	14.4 ± 1.3	2.0 ± 0.1	1.7 ± 0.2	43.6 ± 0.3
TRV *chs/la*-*1*	125	31.1 ± 1.6	22.7 ± 2.1	16.2 ± 2.1	1.9 ± 0.2	1.4 ± 0.2	43.1 ± 0.3

Each data point is the mean (± SD). The statistic significances were examined using student *t* test. The statistic significances of measurements between these two groups are indicated by number of stars. A diagram showing the measured regions is in Additional file 2: Fig.  S2

n, number of lobes; **: p-value < 0.005; ***: p-value < 0.0005; W, lobe width; D, distance between sinuses; L, lobe length

In addition to measurements in corolla limbs, we measured their corolla tube length, tube smallest width, and tube largest width. In these measurements, we did not find any significant differences between compared two groups (data not shown).

### Silencing of *PhLA* affecting epidermal cell shapes in corolla limb

**Fig. 4:**
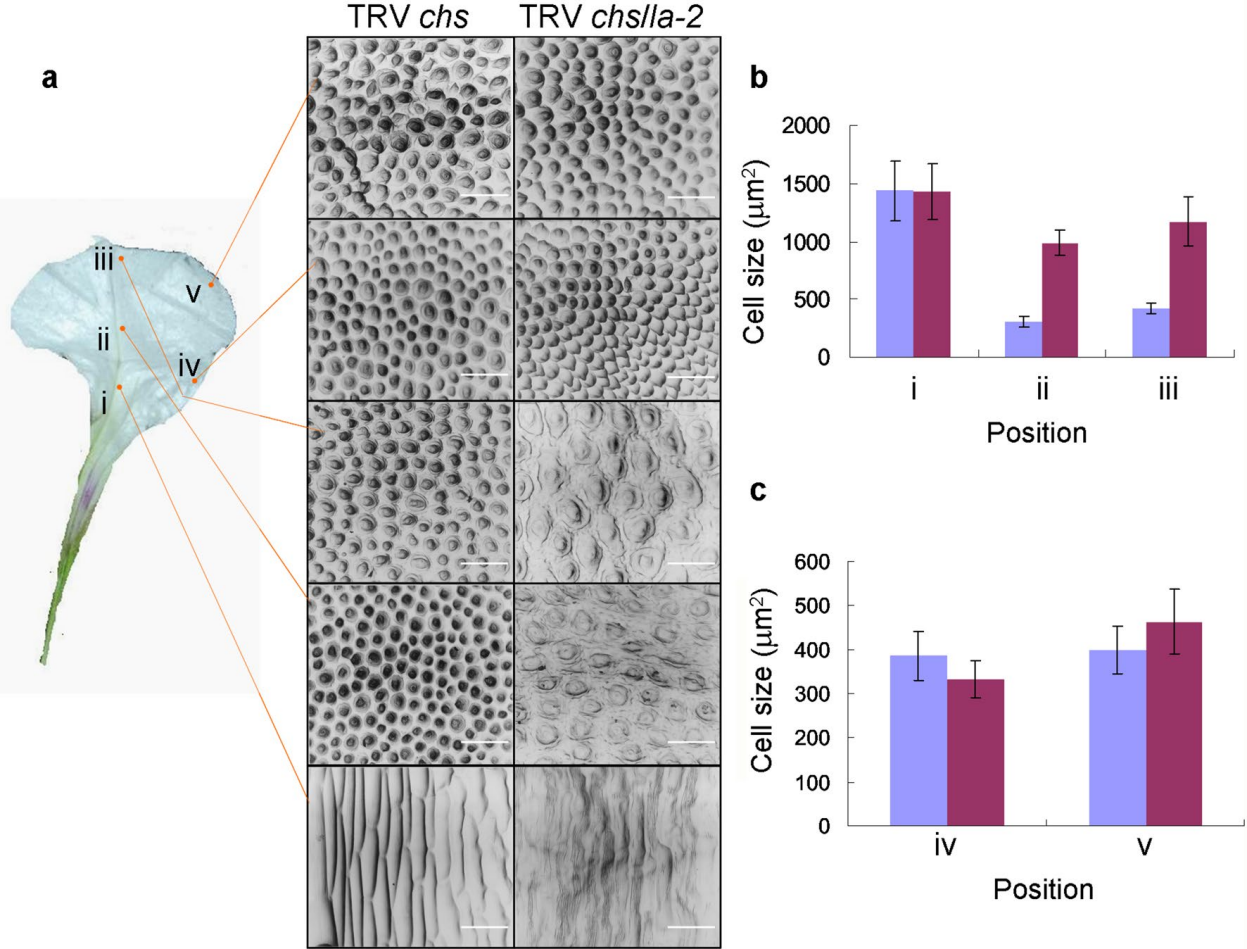
Effects of silencing *PhLA* on petal epidermal cells. **a** SEM micrograph of petal epidermal cells from white flowers of TRV *chs* infected plants compared to those of TRV *chs*/*la*-*2* infected plants. Five regions (i–v) on a lobe from both groups of flowers were examined. A petal segment was dissected to illustrate the five regions. Scar bars: 50 μm. **b** Cell size comparison between petal lobes infected with TRV *chs* and those infected with TRV *chs*/*la*-*2* at the regions along mid-ribs (i–iii). **c** Cell size comparison between petal lobes infected with TRV *chs* and those infected with TRV *chs*/*la*-*2* at the regions on margins (iv–v). Blue bars in (**b**, **c**) indicate the cell sizes from the TRV *chs* group, and purple bars indicate those from the TRV *chs*/*la*-*2* group

The changes in petal lobe shape can result from changes in petal cell shapes or cell numbers, and mutations on *CINCINNATA* in *Antirrhinum* gave rise to flat petal epidermal cells and also reduced cell proliferation in petals (Crawford et al. 2004). In addition, the reflexed corolla curvature in petunia *phmyb1* mutant was associated with defect in petal conical epidermal cell formation (Baumann et al. 2007). To see whether changes in cellular level may account for overall shape changes in *PhLA* silenced corollas, we investigated the effect of *PhLA* silencing on petal epidermal cell formation. Adaxial epidermal cells of petals were observed using scanning electron microscopy (SEM). Five regions in a lobe were checked in which three were picked along the midrib and two were on the side (Fig. 4). The SEM micrographs showed that epidermal cells of *PhLA* silenced flowers in the regions along the midrib (region i, ii, and iii) became flat and their base became irregular; however, the cells on the side (region vi and v) were remained the same (Fig. 4a). Along the midrib, cell sizes at region i of *PhLA* silenced corollas were no significantly different from those of control corollas; however, the sizes became much larger in the *PhLA* silenced group than those in the control group (Fig. 4b). At the region iii, cell sizes in the *PhLA* silenced group were two fold larger than those in the control group. In contrast, on the side of corolla lobes with *PhLA* silenced, cell sizes at both regions measured remained unchanged when compared to corolla lobes without *PhLA* silenced (Fig. 4c).

### Expression of *PhLA* in wild-type corollas

**Fig. 5:**
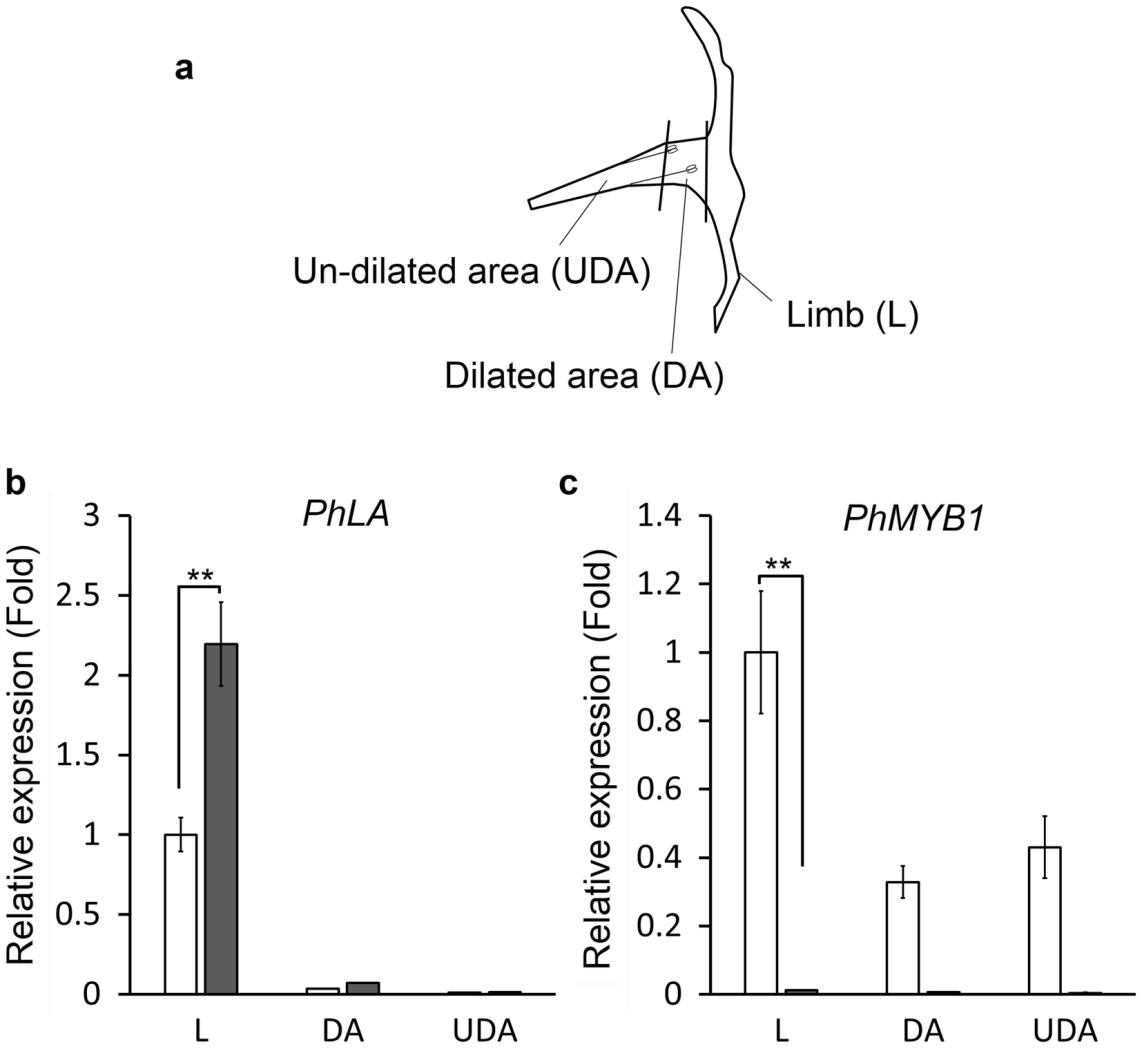
Expression of *PhLA* in fully open flowers. **a** Diagram of a petunia corolla showing three divided regions in the expression analysis. **b** Relative expression of PhLA in different flower regions of W115 (gray bars) or Primetime Blue (white bars) flowers. **c** Relative expression of *PhMYB1* in different flower regions of W115 (gray bars) or Primetime Blue (white bars) flowers. Transcript abundances of *PhLA* and *PhMYB1* are relative to those in limb regions of Primetime Blue flowers. The data show the mean ± SEM from three biological replicates. Expression of *ACTIN* gene was used as an internal control. *Indicates significant differences (**P < 0.005)

**Fig. 6:**
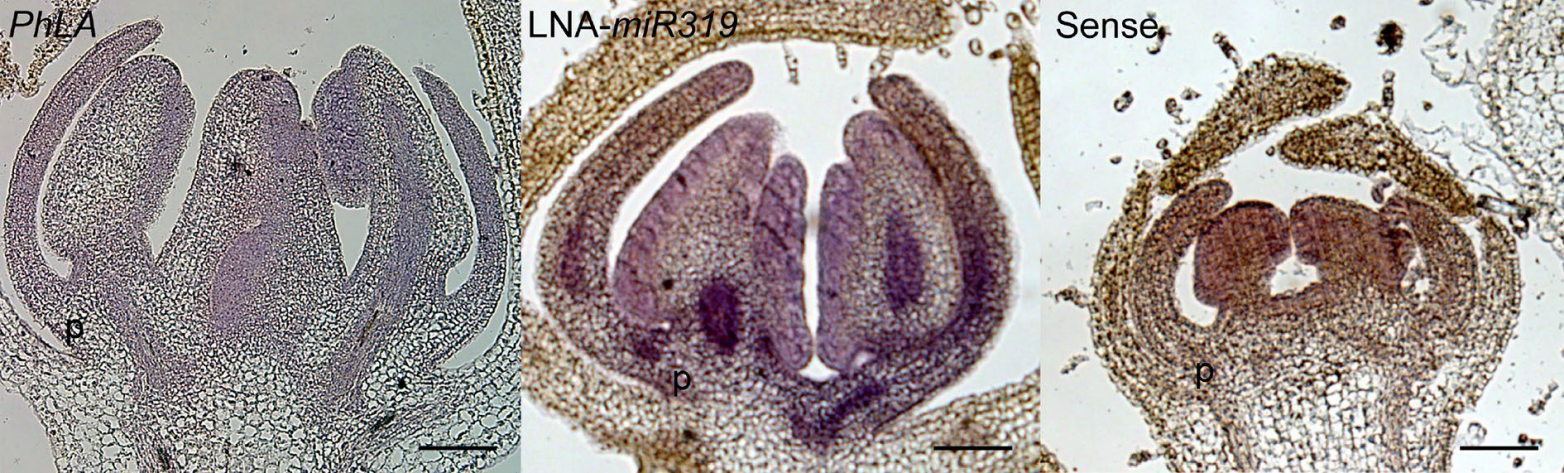
Spatial distribution of *PhLA* and *miR319* in petunia flowers. In situ hybridization of longitudinal sections of wild-type petunia flowers. Sections of flowers were probed with *PhLA* antisense (left panel) or LNA-*miR319* probe (central panel). *PhLA* sense probe (right panel) was used to show the background signals. p, petal; Scar bars: 100 μm

Since the morphological changes caused by *PhLA* silencing were restricted in corolla limbs, we investigated the expression patterns of *PhLA* in corollas at different developmental stages and regions. We divided a corolla into three parts, limb, dilated area, and un-dilated area (Fig. 5a), and analyzed the transcript abundance of *PhLA* in these parts. Without surprise, the majority of *PhLA* transcripts were detected at corolla limbs in two petunia cultivars tested in which W115 corolla tubes are long and narrow while Primetime Blue corolla tubes are short and wide (Fig. 5b). Expression profile of *PhMYB1* was similar to that of *PhLA* with highest expression at the limb regions in both cultivars (Fig. 5c). For the developmental stages, corollas were collected from different sizes of flower buds from 10 to 15 mm buds to fully open flowers. We found that *PhLA* was constantly expressed throughout the developmental stages in both tested cultivars (Additional file 3: Fig. S3). In situ hybridization further confirmed that *PhLA* transcripts were mostly accumulated at the distal regions of petals from early developmental stages (Fig. 6).

Many *CIN*-*TCPs* are post-transcriptionally regulated by *miR319*, including *LANCEOLATE* (Ori et al. 2007). The *PhLA* transcript also contains a *miR319* recognition site (Fig. 1a), and therefore, it is highly likely regulated by *miR319*. To explore whether there is a mutually exclusive expression pattern between *PhLA* and *miR319*, LNA-*miR319* antisense oligo probe was used to detect *miR319* in petunia flowers. Indeed, the strongest signal was detected at proximal site of petals for *miR319*, especially at the rid region of a petal (Fig. 6).

## Discussion

### *LA*-like genes play divergent roles in shaping plant organs in different Solanaceae species

LANCEOLATE promotes leaf maturation. Timing and location of its expression determine the complexity of tomato leaves (Ori et al. 2007; Shleizer-Burko et al. 2011). Our findings also suggest that differential dynamics of *LA* activity are corresponding to the growth and maturation of other Solanaceae species as well. The timing and LA expression levels are related to leaf initiation and morphogenesis in eggplant, pepper, and potato (Shleizer-Burko et al. 2011). The low level expression of *LA* in pepper, a simple leaf species, is correlated with the long initiation stage of its leaf development while eggplant leaves, which show early rise of *LA* expression, exhibit relatively short initiation and primary morphogenesis stages (Shleizer-Burko et al. 2011). This strong correlation suggests that LA plays a pivotal role in leaf morphogenesis.

We used a conserved region of *PhLA* to silence *LA*-like genes in petunia, *N. benthamiana*, and tomato. Since the fragment shares 91% identity with tomato *LANCEOLATE*, and it was suggested that a 24 nt fragment with perfect match to target transcripts is sufficient to trigger gene silencing (Lu et al. 2003), *LA*-like genes should be the primary targets of our VIGS designs. Indeed, an enhanced complexity in tomato leaves was observed and we also found enhanced leaf curvature and expanded leaf margins in *N. benthamiana* after VIGS (Fig. 2b, c). These results are in agreement with the proposed role of *LA* in leaf maturation. However, leaf morphology was not altered after *PhLA* was significantly knockdown in petunia though corolla curvature was enhanced (Figs. 2a, 3a & b). The similar corolla morphological modification was seen also in *N. benthamiana* after VIGS (Additional file 1: Fig. S1). In contrast, no visible floral morphological changes were observed in tomato after VIGS. A gene specific region of *PhLA* was also able to trigger gene silencing and cause morphological changes in petunia corollas (Fig. 3a & b). The findings indicate that PhLA is essential for proper petal morphogenesis but may not be essential for leaf development; however, tomato LA may have a more defined role in leaf morphogenesis, and proper development of leaf and flower requires LA in *N. benthamiana*. It is also possible that other TCP proteins have redundant functions with PhLA on leaf morphogenesis. The functional redundancy has been found in Arabidopsis in which mutations on single CIN-TCP exhibit very mild phenotypic changes in leaves (Schommer et al. 2008) but when expression of multiple members of this group was suppressed, the leaves became extremely large and serrated (Efroni et al. 2008). Taking together, even though *LA* orthologs share high sequence identities, they may be differentially recruited to control different developmental processes in different Solanaceae species.

### PhLA regulates petunia corolla shape independently from PhMYB1

Silencing of *PhLA* resulted in reflexed corolla (Fig. 3a) and flatted epidermal cells (Fig. 4a) that resembles phenotype of *phmyb1* (Baumann et al. 2007). The expression of *PhMYB1*, however, was not affected when a gene specific region was used for VIGS (Fig. 3b). Transcript abundance of *PhLA* is higher in flowers than in leaves while *PhMYB1* is very abundantly expressed in leaves (Fig. 3b, c). Thus, PhLA does not seem to regulate *PhMYB1* at a transcriptional level. A similar result was found in *Antirrhinum*. Both CINCINNATA and MIXTA are required for conical epidermal cell formation in *Antirrhinum* while MIXTA transcript abundance was not affected in *cin* mutants (Crawford et al. 2004). When a conserved sequence was used to silence *PhLA*, transcript abundance of *PhMYB1* was significantly downregulated only in leaves but not in flowers (Fig. 3c). The result may suggest that expression of other *LA*-like genes are affected when a conserved domain is used for VIGS.

### PhLA regulates petal epidermal cell differentiation and corolla limb curvature

Flower shape, color, and texture are important features in attracting animal pollinators (Whitney and Glover 2007), and PhLA is very likely participated in regulation of these important features. The most visible fact caused by *PhLA* silencing was the reflexed petal curvature. To investigate the possible reasons causing the shape alteration, we examined the changes in petal lobes, and found that the proximal sides of petal lobes became narrower while their distal sides further expanded in a *PhLA* silenced flower (Table 1). Surface curvature can be expressed using Gaussian curvature (Nath et al. 2003). When a surface is evenly expanded, a flat surface is maintained and the Gaussian curvature is zero. In contrast, in our case, the margin of a corolla limb was expended faster than the central region, and as a result, a negative curvature was generated. The examination on cellular level confirmed the idea. In the five areas examined in petunia corollas, the cell sizes at the distal side along the midribs (ii & iii) were substantially larger in *PhLA* silenced corollas while the sizes at the proximal side (i) remained unchanged (Fig. 4). This result indicates that the expansion on the distal sides of lobes in *PhLA* silenced corolla was most likely due to enlargement of cell size but not enhancement of cell proliferation. Despite the expansion of distal side of petal lobes, area sizes of the lobes remained unchanged. Therefore, the overall cell number of *PhLA* silenced corollas should be less than that of corollas without *PhLA* silencing. In addition, the cell sizes and shapes on the side (iv & v) of a petal lobe were not changed (Fig. 4). Mutations in *CIN* of *Antirrhinum* also resulted in flatter epidermal cells, but the effect was stronger in edges of dorsal petals but cells close to midribs of ventral petals were less affected (Crawford et al. 2004). Therefore, PhLA and CIN may play related yet different roles in petal morphogenesis. Transgenic *Cyclamen* with ectopically expressed chimeric cyclamen TCP repressor produced flowers with crinkly edges (Tanaka et al. 2011). In addition, transgenic tomato with a *miR319* driven by *FIL* promoter produced flowers with increased curvature and also crinkly edges (Ori et al. 2007). Therefore, a pronounced morphological change on petal edges may be observed when activity of multiple CIN-TCPs is suppressed.

Both PhLA and PhMYB1 are important for petal conical epidermal cell formation. It was shown that alterations on the epidermal cell shapes change overall presentation of corollas (Baumann et al. 2007). In addition, similar to *mixta* mutants in *Antirrhinum*, petals of *phmyb1* are also paler. The color change is due to a difference of light reflection between conical cells and flat cells (Baumann et al. 2007; Noda et al. 1994). The visual effects of conical cells to help get attention from pollinators have been considered to be a reason why many Angiosperm species have evolved flowers with conical epidermal cells (Kay et al. 1981; Whitney et al. 2011). Though *Antirrhinum* flowers carrying *mixta* alleles are indeed less visited by their nature pollinators, bumblebees, than wild-type flowers, the enhanced pollination success resulted from conical cells can still be observed when white flowers were compared (Glover and Martin 1998). It was then showed that the conical-shape of petal cells help pollinators to grip on flowers (Whitney et al. 2009). The changes in epidermal cell shapes may affect pollinator preference due to petal reflexing, which reduces the visual sizes of flowers (Baumann et al. 2007). Indeed, hawkmoths prefer to visit flowers with large corolla limbs (Venail et al. 2010). The evidence provides new aspects for importance of CIN-TCPs in regulation on petal epidermal cell differentiation and petal morphogenesis that may be important for pollinator attraction. The overall expression patterns of both *PhLA* and *PhMYB1* are similar in the two flowers with different shapes in which the flowers of Primetime Blue have a short and wide tube while tubes of W115 flowers are long and narrow (Fig. 5). The transcripts of both *PhLA* and *PhMYB1* are most abundant in the limb regions; however, transcript abundance of *PhLA* is significantly higher in W115 flower than in Primetime Blue flowers while transcript abundance of *PhMYB1* in Primetime Blue flowers is more than 100 folds of that in W115 flowers (Fig. 5). It should be interesting to know whether the different levels of expression in these two genes contribute to flower shape regulation or not.

### Mutually excluded spatial distribution between *PhLA* transcripts and *miR319* during flower development

Similar to tomato *LA*, the *PhLA* transcript contains a *miR319* recognition site, and therefore, is a potential target of this miRNA. In tomato, differential spatial distribution of *LA* and *miR319* was observed during early leaf development in tomato (Ori et al. 2007). However, *miR319* did not completely exclude the expression of *LA*, and overlapping expression domains of both can be found at shoot apical meristem, young leaf primordia and developing leaflets. Therefore, *miR319* serves a modulator to fine tune timing and location of *LA* expression (Ori et al. 2007). *PhLA* is predominately expressed in corolla limbs (Fig. 5). We, therefore, explored the potential spatial regulation of *PhLA* by *miR319*. In situ hybridization revealed the potential spatial antagonic regulation (Fig. 6). Similar to the findings in tomato leaf development, *PhLA* and *miR319* are differentially expressed in developing petunia corollas, and overlapping expression regions were also found. Therefore, *miR319* should also play a role as a CIN-TCP modulator in regulating petal curvature.

## Conclusions

Petunia *LANCEOLATE* ortholog, *PhLA*, may have a more define role in shaping petunia flower. Silencing of *PhLA* clearly enhances the curvature of petunia corolla lobes, but shows no visible effect on leaf morphology. The enhanced petal curvature is likely due to defects of conical epidermal cell formation, and the alteration of petal curvature and epidermal cell shape may affect pollinator attraction of the flower. Our results demonstrate the importance of LA-like activity to morphological regulation in Solanaceae species, and also show that VIGS is a powerful technique in functional studies in close species.

## Supplementary information

**Additional file 1: Figure S1.** Effects of silencing *LA*-like genes on *Nicotiana benthamiana* flower. (a) Front view and (b) side view of *N. benthamiana* flowers with or without *LA*-like genes silenced. Flowers infected with TRV *chs*/*la-1*, which contains a conserved fragment of *PhLA* cDNA, shows reflexed corolla lobes.

**Additional file 2: Figure S2.** Schematic representation of a petunia flower. The scheme shows the locations of measurements indicated in Table 1. W: lobe width; L: lobe length; D: Distance between sinuses.

**Additional file 3: Figure S3.** Expression of *PhLA* during corolla development. Expression profiles of *PhLA* in two different petunia cultivars, W115 and Primetime Blue, during their corolla elongation were examined. Corolla sizes (1, 0–1 cm; 2, 1–2 cm; 3, 2–3 cm; 4, 3–4 cm; 5, open flower for Primetime Blue and 4–5 cm for W115; 6, open flower for W115).

## Data Availability

All of the data and materials are available upon request.
