# Supplementary figures and images for: Silencing of PhLA, a CIN-TCP gene, causes defected petal conical epidermal cell formation and results in reflexed corolla lobes in petunia
Source: Bot Stud. 2020 Sep 17;61:24. doi: 10.1186/s40529-020-00300-7 (PMC7498528; doi:10.1186/s40529-020-00300-7)

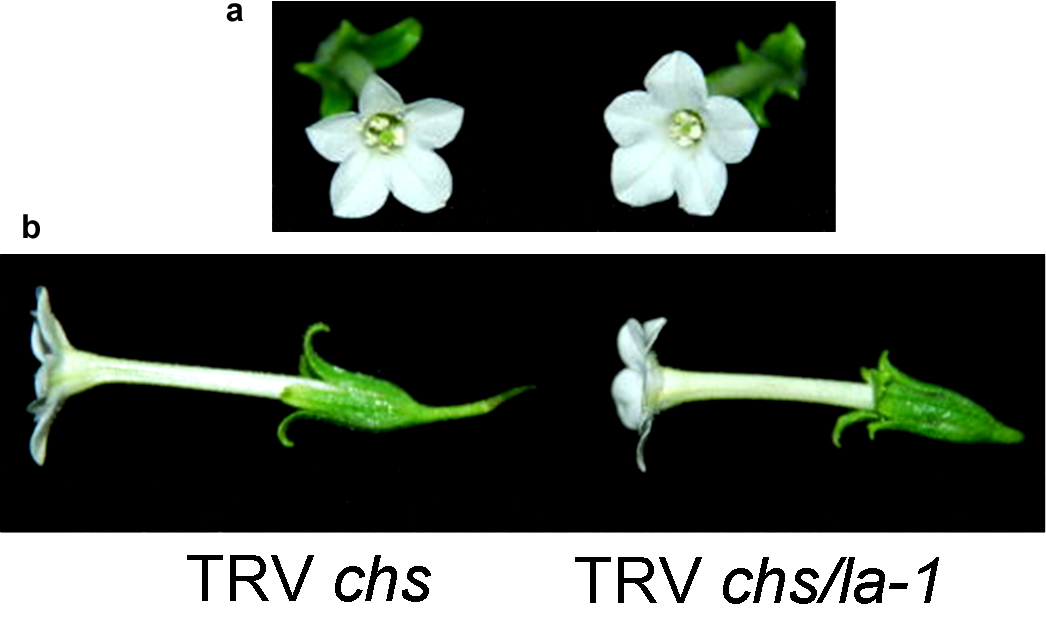

Supplement: Supplementary file 1 — Additional file 1: Figure S1. Effects of silencing LA-like genes on Nicotiana benthamiana flower. (a) Front view and (b) side view of N. benthamiana flowers with or without LA-like genes silenced. Flowers infected with TRV chs/la-1, which contains a conserved fragment of PhLA cDNA, shows reflexed corolla lobes. [file 40529_2020_300_MOESM1_ESM.tif]

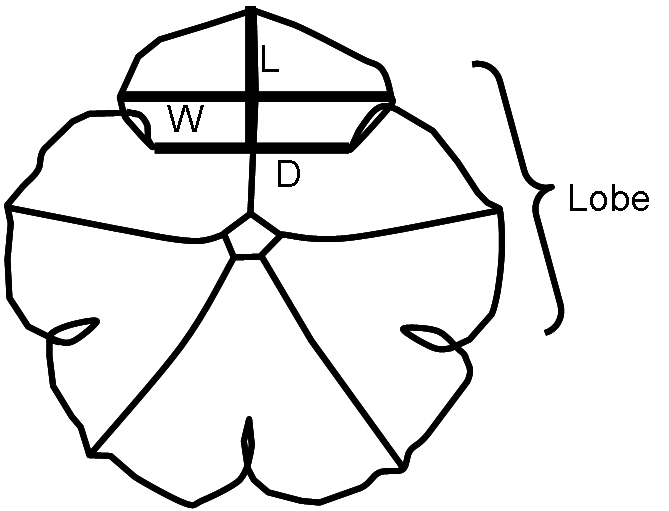

Supplement: Supplementary file 2 — Additional file 2: Figure S2. Schematic representation of a petunia flower. The scheme shows the locations of measurements indicated in Table 1. W: lobe width; L: lobe length; D: Distance between sinuses. [file 40529_2020_300_MOESM2_ESM.tif]

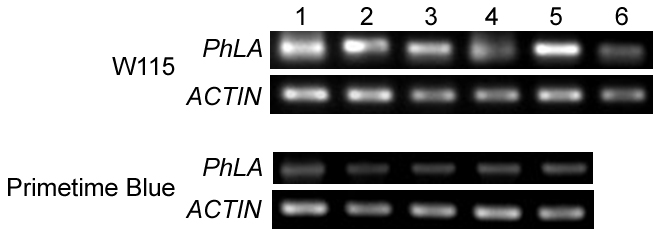

Supplement: Supplementary file 3 — Additional file 3: Figure S3. Expression of PhLA during corolla development. Expression profiles of PhLA in two different petunia cultivars, W115 and Primetime Blue, during their corolla elongation were examined. Corolla sizes (1, 0–1 cm; 2, 1–2 cm; 3, 2–3 cm; 4, 3–4 cm; 5, open flower for Primetime Blue and 4–5 cm for W115; 6, open flower for W115). [file 40529_2020_300_MOESM3_ESM.tif]
